# Rapid Determination of 12 Classes of Per- and Polyfluoroalkyl Substances in Water Samples from Environmental Forensic Cases

**DOI:** 10.3390/molecules29163881

**Published:** 2024-08-16

**Authors:** Bing Li, Meihui Wang, Kuan Cheng, Xueyan Guo, Ruyin Dong, Keming Yun, Dong Ma

**Affiliations:** 1 Shanxi Key Laboratory of Forensic Medicine, Key Laboratory of Forensic Toxicology of Ministry of Public Security, School of Forensic Medicine, Shanxi Medical University, Jinzhong 030600, China; libing_0706@163.com (B.L.); 13210798295@163.com (M.W.); 2 Key Laboratory of Forensic Science, Ministry of Justice, Shanghai Forensic Service Platform, Academy of Forensic Science, Shanghai 200063, China; chengk@ssfjd.cn (K.C.); guoxy@ssfjd.cn (X.G.); dongry@ssfjd.cn (R.D.)

**Keywords:** per- and polyfluoroalkyl substances, surface water, groundwater, wastewater, simple sample preparation, Orbitrap

## Abstract

The widespread use of per- and polyfluoroalkyl substances (PFASs) with different physico–chemical properties poses a great threat to the environment and human health. Simultaneous detection of different classes of PFASs is a difficult task, especially for rapid analysis of polluted water samples in environmental forensic cases. In this study, a simple sample preparation ultrahigh-performance liquid chromatography coupled with quadrupole Orbitrap high-resolution mass spectrometry was established for the detection of PFASs in a wide range of water matrices. By optimizing the conditions of pretreatment and the parameters of the instrument, the developed method provided good linearity of calibration standards (R^2^ > 0.99), and demonstrated excellent MLOQ (0.008–1.2 µg/L), with spiked recoveries ranging from 57.7% to 151% for 47 targets in surface water samples, and from 45.7 to 165% for 46 targets in ground and waste water samples, respectively. This method required an injection volume of 3 µL and an analysis time of only 18 min per sample. The validation method was successfully applied to the analysis of 20 environmental water samples, in which 15 target substances with different concentrations were detected, with total concentrations of 0.082 to 262.455 μg/L. The method is simple and exclusive, and can rapidly confirm the occurrence of PFASs in different water samples, providing a convenient and fast high-throughput analysis, which is especially suitable for the application in the environmental forensic investigation of PFASs pollution.

## 1. Introduction

Per- and polyfluoroalkyl substances (PFASs) have attracted significant attention as “persistent organic pollutants” over the past 20 years, with more than 12,000 chemicals in the CompTox Chemicals Dashboard [1]. The definition of PFASs has recently been revised by the Organization for Economic Co-operation and Development (OECD) and refers to a group of fluorinated substances that contain at least one fully fluorinated methyl or methylene carbon (without any H/Cl/Br/I atoms attached) [2]. The strong C-F bond of these molecules results in chemical stability, water and oil repellency, thermal stability and surfactant properties, which have led to their application in over 200 products or industries. The main application areas include the following: surfactants, emulsifiers, lubricants, firefighting foams, fast food packaging, carpets, cosmetics, clothes, dental floss, and other commonplace items [3,4].

Dry and wet atmospheric deposition, soil and street surface runoff, landfill leachate, industrial sources and municipal wastewater treatment plants are the most important sources of PFASs pollution [5]. At present, there are two views on the emergence of PFASs in environmental media: one is the long-distance transport of semi-volatile and volatile PFASs with atmospheric motion [6], and the other is the migration of ionic PFASs driven by water circulation [7]. PFASs not only negatively impact the environment, but human exposure to them can also lead to various health issues, including immune system disorders, endocrine disruptions, kidney problems, obesity, low birth weight, asthma, allergies, and an increased risk of cancer [8,9]. Because of the harm to the environment and human health, typical PFASs, for example, perfluorooctanoic acid (PFOA), perfluorooctane sulfonate (PFOS), and perfluorohexane sulfonate (PFHxS), were successively included in the list of the Stockholm Convention Persistent Organic Pollutants (POPs) [10]. Long-chain perfluorocarboxylic acids (PFCAs), their salts, and related compounds came under scrutiny as the latest persistent organic pollutant candidates in 2021 [11]. China’s Ministry of Ecology and Environment and five other departments have also included PFOA, PFOS, PFHxS, their salts, and related compounds in the list of key control of emerging pollutants (2023 version) [12].

With the restricted use of legacy long–chain PFASs, alternatives such as perfluorobutanoic acid (PFBA), perfluorobutyl sulfonate (PFBuS), hexafluoropropylene oxide dimer acid (GenX), chlorinated polyfluorinated ether sulfonic acids (F-53B) and others have flooded the market. At present, studies have proved that such emerging alternatives are also environmentally persistent and biotoxic, and they have been widely detected in the surface, ground, waste and drinking water at concentrations ranging from ng/L to low mg/L levels [13,14,15,16,17,18]. Establishing rapid and efficient analytical methods to quantify and identify legacy and emerging PFASs is essential to study their environmental behavior and hazardous effects.

Employing solid-phase extraction (SPE) and ultrahigh-performance liquid chromatography (UHPLC) coupled with tandem mass spectrometry (MS/MS) is currently the most widely used analytical approach for the quantification of PFASs. SPE can improve the sensitivity of the method, especially when the target substances are at low to mid ng/L concentrations [19,20,21,22,23,24,25]. Nevertheless, it has limitations for high-throughput environmental forensic analysis. In such pollution cases, the concentrations of PFASs in the water samples exceeded µg/L levels. Not only should sensitivity and accuracy be improved, but the method should reduce the time required for sample preparation and instrumental analysis. The development of SPE for a wide range of target PFASs is a complex process, involving the optimization of solvent conditions at each stage. This process can be laborious, time-consuming and impractical for large-scale monitoring activities [26]. In addition, the physico-chemical properties of PFASs greatly vary depending on the chain length and the acidic group present in the molecule, thus making challenging the recoveries of all analytes during extraction and clean-up processes [27]. In addition, PFASs are often found in laboratory supplies, and excessive pretreatment processes can cause the PFASs to dissolve out of them, resulting in false positive results.

In addition, the volume of water samples in environmental forensic cases is usually small, the samples are not reproducible, and the size of samples used should be minimized at the testing stage to avoid wasting if the test fails. The methods based on SPE [16,20,28,29] have a sample volume of 100 mL or even 2000 mL. A new method using small amounts is preferred over one involving SPE.

Therefore, new analytical approaches that offer easy sample preparation and sensitive detection are attractive to the community of researchers on PFASs and environmental forensic investigation due to high concentrations of PFASs pollution.

High-resolution mass spectrometry (HRMS) demonstrates significant potential for both accurate mass measurement and characteristic fragment identification [20], making simple sample preparation an effective approach. Minimizing sample preparation is optimal for mitigating contamination arising from manipulation and treatment, while also ensuring high analytical throughput. In this study, we present a simple, rapid, sensitive, and environmentally friendly method based on UHPLC–quadrupole Orbitrap HRMS for the simultaneous determination of 12 classes of target PFASs in various environmental water samples. This method does not require large volume injections or complex pretreatment procedures, thereby facilitating rapid environmental forensic investigations of PFASs pollution.

## 2. Results and Discussion

### 2.1. Optimization of Instrumental Parameters

#### 2.1.1. Optimization of UHPLC Parameters

**Figure 1:**
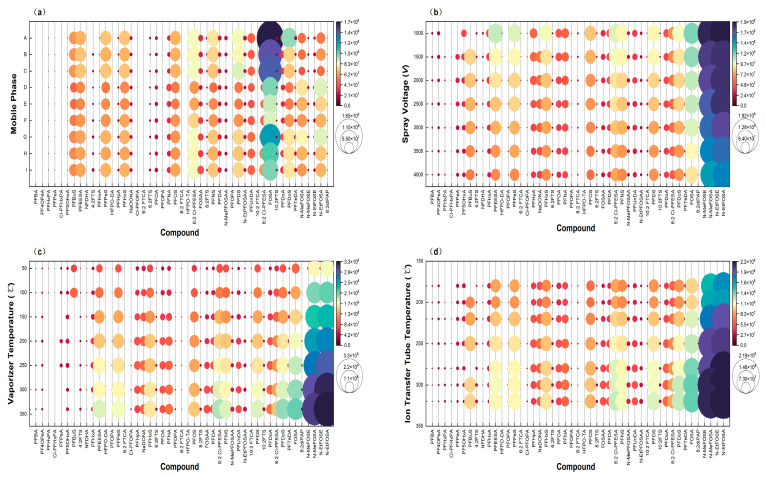
(**a**) Comparison of the responses of target compounds in different mobile phases. A: 2 mM NH_4_OAc in H_2_O–MeOH; B: 5 mM NH_4_OAc in H_2_O–MeOH; C: 10 mM NH_4_OAc in H_2_O–MeOH; D: 2 mM NH_4_OAc in H_2_O–ACN; E: 5 mM NH_4_OAc in H_2_O–ACN; F: 10 mM NH_4_OAc in H_2_O–ACN; G: 2 mM NH_4_OAc in H_2_O–MeOH and ACN (*v:v* = 1:1); H: 5 mM NH_4_OAc in H_2_O–MeOH and ACN (*v:v* = 1:1); I: 10 mM NH_4_OAc in H_2_O–MeOH and ACN (*v:v* = 1:1). (**b**–**d**) Spray voltage, vaporizer and ion transfer tube temperature optimization for 47 target compounds.

UHPLC basic conditions include mobile phase composition, column selection, and mobile phase gradient. According to current research on PFASs detection, NH_4_OAc is often used as an additive in the mobile phase A to improve sensitivity and peak shape. As for the mobile phase B, MeOH and ACN are commonly selected [19,21,28]. In this study, nine mobile phase compositions were tested: 2 mM NH_4_OAc in H_2_O–MeOH, 5 mM NH_4_OAc in H_2_O–MeOH, 10 mM NH_4_OAc in H_2_O–MeOH, 2 mM NH_4_OAc in H_2_O–ACN, 5 mM NH_4_OAc in H_2_O–ACN, 10 mM NH_4_OAc in H_2_O–ACN, 2 mM NH_4_OAc in H_2_O–MeOH and ACN (*v:v* = 1:1), 5 mM NH_4_OAc in H_2_O–MeOH and ACN (*v:v* = 1:1), and 10 mM NH_4_OAc in H_2_O–MeOH and ACN (*v:v* = 1:1) (Figure 1a). The results showed that the addition of NH_4_OAc significantly improves the peak shape of almost all compounds. The mixture of MeOH and ACN (*v:v* = 1:1) had better elution performance than MeOH or ACN alone and could reduce background interference in the MS detector. Due to the comprehensive consideration of sensitivity, retention time, chromatographic separation and peak type, 2 mM NH_4_OAc in H_2_O–MeOH and ACN (*v:v* = 1:1) were finally selected as the mobile phase.

**Figure 2:**
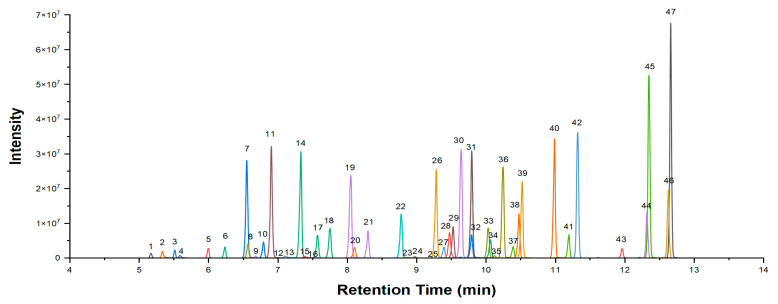
The typical chromatograms of 47 target PFASs in a single injection of 5 µg/L standards. 1. PFBA; 2. PFHxPA; 3. PF4OPeA; 4. Cl–PFHxPA; 5. PFPeA; 6. PF5OHxA; 7. PFBuS; 8. 4:2FTS; 9. NFDHA; 10. PFHxA; 11. PFEESA; 12. HFPO–DA; 13. PFOPA; 14. PFPeS; 15. Cl–PFOPA; 16. 6:2 FTCA; 17. PFHpA; 18. NaDONA; 19. PFHxS; 20. 6:2FTS; 21. PFOA; 22. PFNA; 23. PFDPA; 24. 8:2 FTCA; 25. HFPO–TA; 26. PFOS; 27. 8:2FTS; 28. FOSAA; 29. PFDA; 30. 6:2 Cl–PFESA; 31. PFNS; 32. N–MePFOSAA; 33. PFUnDA; 34. N–EtPFOSAA; 35. 10:2 FTCA; 36. PFDS; 37. 10:2FTS; 38. PFDoA; 39. 8:2 Cl–PFESA; 40. PFDoS; 41. PFTeDA; 42. FOSA; 43. 8:2diPAP; 44. N–MeFOSE; 45. N–MeFOSA; 46. N–EtFOSE; 47. N–EtFOSA.

Due to the different properties of the PFASs of interest, better separation after eluting through the column is a key factor in ensuring accurate analysis. Therefore, four chromatographic columns were tested in this work. The responses and specifications of the target compounds of the four columns are shown in Figure S1 and Table S2. The mobile phase gradients were also optimized (Figure S2). The chromatograms of PFASs obtained under optimized conditions are shown in Figure 2.

#### 2.1.2. Optimization of HRMS Parameters

The optimization of HRMS parameters is also particularly important for improving the responses of the target compounds. Spray voltage, vaporizer and ion transfer tube temperatures were examined (Figure 1b–d). When one parameter was being optimized, the others remained unchanged. After obtaining the best instrument response conditions, the full scan accurate mass was the quantitative ion. Because the mobile phase contains NH_4_OAc, N-MeFOSE and N–EtFOSE can form [M+CH_3_COO]^−^ with acetate ions; for all the remaining substances, the quantitative ions are [M−H]^−^ [22,28]. The quantitative ions of PFASs are shown in Table 1.

### 2.2. Optimization of Sample Pretreatment

#### 2.2.1. Evaluation of Solvent Matrix Effects

In order to avoid solvent matrix effects and reduce the influence on the measurement results, different proportions of MeOH:water solution were compared according to what is commonly used. The compounds were diluted with MeOH:water solution (*v:v* = 3:7, *v:v* = 5:5, *v:v* = 7:3) and pure MeOH, respectively, and then analyzed on the instrument. Comparing the response values of each substance, it was found that the matrix effect of the solvent had a greater influence on the results, and the response of MeOH:water solution was significantly better than that of pure MeOH (Figure S3). Considering the response, peak shape and other factors, MeOH:water solution (*v:v* = 5:5) was finally selected as the solvent to dilute the working standard solution for preparing the samples at each concentration of the linear range.

**Figure 3:**
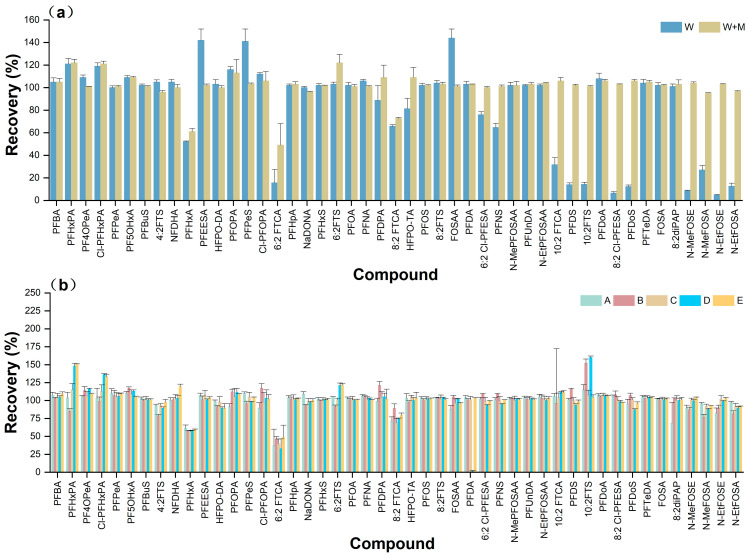
(**a**) Effect of MeOH on spiking recovery. W: water group; W + M: MeOH:water solution (*v:v* = 5:5) group. (**b**) Effect of filter on pretreatment recovery. A: PTFE filters (17 mm × 0.2 μm); B: CA filters (17 mm × 0.2 μm); C: GMF filters (30 mm × 3.1 μm); D: Nylon filters (17 mm × 0.2 μm); E: Nylon filters (30 mm × 0.2 μm).

In order to ensure that the conditions of the samples tested were consistent with the conditions at each concentration point of the working curve, the effect of adding an equal volume of MeOH to the water sample on recovery was verified during the pretreatment phase (Figure 3a). The addition of MeOH significantly improved the spiking recoveries of most of the substances; therefore, in the pretreatment phase, it was necessary to add an equal volume of MeOH to make a mixed sample.

#### 2.2.2. Optimization of Filters

Due to the differences in various types of water samples, the supernatant after pretreatment should be filtered before being tested on the instrument. We compared the effectiveness of the following different filter types for each target (Figure 3b): Nylon filters (17 mm × 0.2 μm), Nylon filters (30 mm × 0.2 μm), Cellulose Acetate filters (CA filters, 17 mm × 0.2 μm), Polytetrafluoroethylene filters (PTFE filters, 17 mm × 0.2 μm), and Glass Microfiber filters (GMF filters, 30 mm × 3.1 μm). Compared to several other filters, PTFE filters had the lowest effect on the recovery of most targets, and were chosen for the experiment.

In order to prevent PFASs background contamination during pretreatment operations and instrumental analysis, solvent blanks and laboratory procedural blanks (ultrapure water extracted according to the same procedure as the samples) were analyzed to assess the initial concentrations of PFAS, all of which were shown to be lower than the LOQ (Table S3).

### 2.3. Method Validation

#### 2.3.1. Selectivity

The selectivity of the method was evaluated using blank samples and revealed no response attributable to the target compounds; the results are displayed in Table S3.

#### 2.3.2. Linearity, Instrumental Limit of Quantification (LOQ), Method Limit of Detection (MLOD), and Method Limit of Quantification (MLOQ)

Eleven concentrations (0.001, 0.005, 0.01, 0.05, 0.1, 0.25, 0.5, 1, 5, 10, 25 µg/L) were prepared with working standard solution to fit the working curves, and 1/x was used as a factor to weight the curves to improve accuracy. The results showed that the correlation coefficient (R^2^) of all 47 targets was greater than 0.99, showing good linearity. Moreover, the developed method demonstrated excellent MLOQ (0.008–1.2 µg/L) (Table 2).

#### 2.3.3. Accuracy, Matrix Spiking Recovery, and Precision

Due to the different MLOQ of each target, the accuracy (matrix spiking recovery) and precision of the method were assessed by adding low (0.05/1 µg/L), medium (5 µg/L) and high concentrations (20 µg/L) PFASs in actual surface, ground, and waste water samples. As presented in Table 3, the accuracy for 47 targets in surface water samples ranged from 57.7% to 151%. Table S4 shows the accuracy and precision for 46 target PFASs in ground and waste water samples. The recoveries of these targets ranged from 45.7 to 165%. Due to matrix effects (signal suppression) resulting in low recoveries of 8:2diPAP in groundwater and wastewater, the detection targets for these two water bodies are not included.

In all water samples, the method demonstrated good intra-day precision (0.024–19%) and inter-day precision (0.33–19%) for PFASs at four concentrations (n = 3).

### 2.4. Application to Water Samples

**Figure 4:**
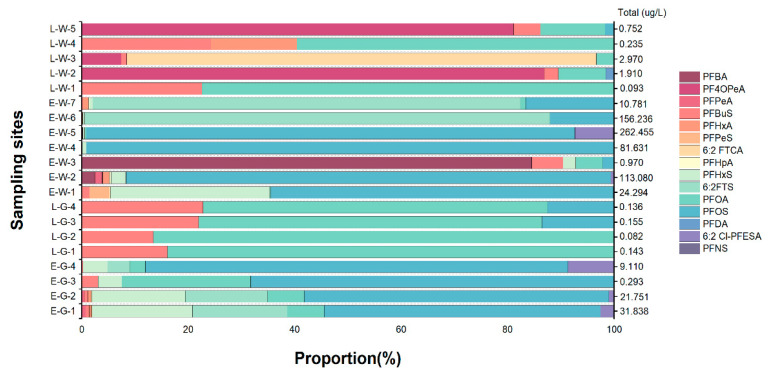
PFASs composition and total PFASs concentration (μg/L) in ground and waste water samples.

The well-optimized method was successfully applied to detect the concentrations of the targets in eight groundwater and twelve wastewater samples from industrial parks of electroplating plants and leather manufacturing factories. A total of fifteen PFASs (PFBA, PF4OPeA, PFPeA, PFBuS, PFHxA, PFPeS, 6:2 FTCA, PFHpA, PFHxS, 6:2FTS, PFOA, PFOS, PFDA, 6:2 Cl–PFESA, PFNS) were detected in varying concentrations (Table S5), with total levels (∑PFASs) ranging from 0.082 to 262.455 μg/L (Figure 4). Previous studies have reported similar concentrations. For example, the discharge of a fluoropolymer production plant caused high levels (from 37.2 to 454,000 ng/L) of PFASs pollution of surface water samples in Xiaoqing River. The target substances were similar to those we detected [30], such as PFBA, PFPeA, PFHxA, PFHpA, PFOA, PFNA, PFDA, PFUnDA, PFBuS, PFHxS, PFOS, PF4OPeA PF5OHxA and others. Sixteen PFASs were found in the wastewater of typical industries in Chongqing, China. The main pollutants in these samples were PFBuS, PFPeA, PFHxA, PFHxS, PFHpA, PFOA, PFOS, PFNA, PFDA, PFDS, PFUnDA, PFDoA, PFTrDA, PFTeDA, PFHxDA, and PFODA, and the ∑PFASs ranged from 0.012 to 38.484 μg/L [31]. Additionally, the concentrations of PFASs in wastewater from a fluoride industrial park near the lower Yangtze River in eastern China varied from 14.7 to 5,200 μg/L [32].

Among these samples, PFCAs and PFSAs were the two most-detected classes, with PFBuS (0.009–0.35 μg/L) and PFOA (0.049–2.21 μg/L, except E-W-4) being found in all samples. PFOS was detected in 70% of the samples, while the remaining contaminants were detected at rates lower than 70%. This suggests that short-chain (≤8) PFASs are widely used in industrial production.

Moreover, PFOA was found at the highest concentration in E-W-5 (240.93 μg/L), while 6:2FTS was also detected at elevated levels (136.44 μg/L) in E-W-6. It is possible that 6:2FTS, as a substitute for PFOS, has been utilized as a novel chromium mist inhibitor in significant amounts within the electroplating sector.

## 3. Materials and Methods

### 3.1. Chemicals and Materials

A total of 47 target PFASs and 20 isotopically labeled analogues used as internal standards (IS) were included in this study. Both the native standards and ISs were purchased from Wellington Laboratories (Guelph, ON, Canada). More detailed information is given in Table S1. The HPLC grade solvents, ammonium acetate (NH_4_OAc), methanol (MeOH) and acetonitrile (ACN) were purchased from Merck (Darmstadt, Germany). Ultrapure water was obtained from a Milli–Q system (0.22 μm filtered, 18.2 mΩ/cm, Millipore, Massachusetts, USA). Nylon filters (17 mm × 0.2 μm), Nylon filters (30 mm × 0.2 μm), CA filters (17 mm × 0.2 μm), PTFE filters (17 mm × 0.2 μm), and GMF filters (30 mm × 3.1 μm) were purchased from Thermo Fisher Scientific (Waltham, MA, USA).

### 3.2. Sample Collection

Surface water samples were collected in stainless steel buckets from a depth of 1 m below the surface. Ground water samples were collected from monitoring wells by bailer tubes. Wastewater samples were collected from wastewater treatment plants (WWTPs) by automatic sampling devices. Before sampling, polypropylene bottles were sequentially washed with MeOH and ultrapure water. Each sample was collected at the volume of 50 mL. The samples were kept at 4 °C for a maximum of 2 weeks prior to analysis.

### 3.3. Sample Pretreatment

All samples were thawed to room temperature and manually shaken before pretreatment. A total of 0.75 mL of each water sample was added to 0.75 mL of MeOH to make a mixed sample for processing. The 990 µL mixed sample was added to a 1.5 mL centrifuge tube. Then the sample was spiked with 10 µL of the 100 µg/L mixed ISs. This was followed by mixing using a vortex shaker for 30 s. The mixture was then centrifuged at 13,400 g for 5 min. Afterwards, the supernatant was filtered through PTFE filters into a polypropylene vial for analysis. Each sample was analyzed in triplicate.

### 3.4. Instrument Analysis

The target PFASs were analyzed by ultrahigh-performance liquid chromatography (Vanquish Flex UHPLC, Thermo Fisher Scientific, Waltham, MA, USA) coupled with quadrupole Orbitrap HRMS (Orbitrap Exploris 120, Thermo fisher Scientific, Waltham, MA, USA). Chromatographic separation was achieved with an Acclaim RSLC C18 column (150 × 2.1 mm, 2.2 μm, Thermo fisher Scientific, Waltham, MA, USA) at a column temperature of 35 °C. A trap column (Hypeisil Gold C18, 50 × 2.1 mm, 3 μm, Thermo fisher Scientific, Waltham, MA, USA) was added between the mixer and the sample injector. Baseline separation was achieved using mobile phases under multistep gradient elution with a total runtime of 18 min at a flow rate of 0.3 mL·min^−1^. The mobile phase A was 2 mM NH_4_OAc water solution; the mobile phase B was MeOH and ACN (*v:v* = 1:1). The mobile gradient was as follows: 10% B for 1 min, from 10% to 45% in 2 min, a linear increase from 45% to 100% between 3 and 11 min, 100% for 4 min, a 10% decrease at 15.01 min, and a final hold to 18 min for system re-equilibration. The injection volume was 3 μL.

The detection and quantification of PFASs were performed under negative heated electrospray-ionization (HESI−) in full Scan mode. The general instrumental conditions were as follows: spray voltage, −1000 V; sheath gas flow rate, 50 arbitrary units (a.u.); aux gas flow rate, 15 a.u.; vaporizer temperature, 340 °C, and ion transfer tube temperature, 320 °C. All the target compounds were monitored within the 5 parts-per-million mass error range (±5 ppm). The mass range for the full scan was set at 150–1300 *m/z* with a resolution of 60,000. The automatic gain control (AGC) target was assigned a value of 200%. X-calibur and Trace Finder 5.1 (Thermo Fisher Scientific, Waltham, MA, USA) were used for both qualitative and quantitative analyses.

### 3.5. Method Validation, Quality Assurance and Quality Control

The method was evaluated in these aspects: selectivity, linearity, LOQ, MLOD, MLOQ, accuracy, matrix spiking recovery, and precision. These were assessed according to the standard “Technical guideline for the development of environmental monitoring analytical method standards” [33] published by the Ministry of Ecology and Environment of the People‘s Republic of China.

#### 3.5.1. Selectivity, Linearity, LOQ, MLOD, and MLOQ

Selectivity characterizes whether an analytical method can distinguish interference of similar compounds and was evaluated by analyzing blank surface, ground, and waste water samples.

In order to effectively reduce the loss of trace pollutants in the pretreatment process, the internal standard method was used for quantitative analysis. Working curves were prepared in MeOH:water solution (*v:v* = 1:1) at 11 levels (0.001, 0.005, 0.01, 0.05, 0.1, 0.25, 0.5, 1, 5, 10, 25 µg/L), with all ISs levels at 1 µg/L.

The LOQ was expressed as the lowest concentration in the linear range. The MLOD and MLOQ were assessed by detecting low concentrations of all compounds spiked in MeOH:water solution (*v:v* = 1:1) at 0.01, 0.05, 0.1, 0.25, 0.5, 1, 2 µg/L, with all ISs levels at 1 µg/L. Following all the steps of pretreatment and analysis, the spiked samples were analyzed by 7 consecutive injections. The MLOD was calculated according to Equation (1):

(1) MLOD=3.143×S

where *S* represents the standard deviation of the 7 measurement results, and the MLOQ was defined as 4 times MLOD.

#### 3.5.2. Accuracy, Matrix Spiking Recovery, and Precision

The accuracy, matrix spiking recovery and precision of the method were assessed by adding low (0.05/1 µg/L), medium (5 µg/L) and high concentrations (20 µg/L) of all analytes to blank surface, ground, and waste water samples. All the samples were analyzed three times a day using the established pretreatment and instrument methods, and the same procedure was performed for three consecutive days, with matrix spiking recovery as accuracy and relative standard deviation (RSD) as precision. The spiking recovery was calculated according to Equation (2):

(2) $$Spiking\;recovery\;(\%)=(\frac{C_{A}-C_{B}}{C_{C}})\times 100$$

where *C_A_* is the concentration of the spiked sample, µg/L; *C_B_* is the background concentration of the sample, µg/L; and *C_C_* is the spiked concentration, µg/L.

## 4. Conclusions

In this study, a simple sample preparation–UHPLC–Orbitrap method was established using a high-sensitivity mass spectrometer to realize the rapid quantification of nearly 50 PFASs in different water matrices. By optimizing the conditions of pretreatment and the parameters of the instrument, the developed method provided good linearity with calibration standards (R^2^ > 0.99) and demonstrated excellent MLOQ (0.008–1.2 µg/L). This method required a sample volume of less than 1 mL and an analysis time of only 18 min per sample. The validation method was successfully applied to the analysis of 20 environmental water samples, in which 15 target substances with different concentrations were detected, with total concentrations from 0.082 to 262.455 μg/L.

The method has several advantages. It involves a simple pretreatment, is environmentally friendly, and has a short analysis time, which can facilitate the detection of PFASs, save analysis time and provide indicative evidence for the environmental forensic investigation of sudden PFASs pollution cases.

## Figures and Tables

**Table 1:** Theoretical and measured (*m*/*z*), mass accuracy errors, and retention times of 47 target PFASs.

Compound	Theoretical Mass (*m/z*)	Measured Mass (*m*/*z*)	Mass Accuracy Error (ppm)	Retention Time (min)
**PFBA**	212.97920	212.97914	−0.3	5.17
**PFPeA**	262.97601	262.97595	−0.2	6
**PFHxA**	312.97281	312.97272	−0.3	6.79
**PFHpA**	362.96962	362.96915	−1.3	7.57
**PFOA**	412.96643	412.96597	−1.1	8.3
**PFNA**	462.96323	462.96283	−0.9	8.77
**PFDA**	512.96004	512.95966	−0.7	9.52
**PFUnDA**	562.95684	562.95667	−0.3	10.03
**PFDoA**	612.95365	612.95300	−1.1	10.47
**PFTeDA**	712.94726	712.94678	−0.7	11.19
**PFBuS**	298.94299	298.94278	−0.7	6.55
**PFPeS**	348.93980	348.93924	−1.6	7.33
**PFHxS**	398.93660	398.93610	−1.3	8.05
**PFOS**	498.93022	498.92978	−0.9	9.28
**PFNS**	548.92702	548.92657	−0.8	9.79
**PFDS**	598.92383	598.92303	−1.3	10.24
**PFDoS**	698.91744	698.91632	−1.6	10.99
**FOSA**	497.94620	497.94565	−1.1	11.32
**N-MeFOSA**	511.96185	511.96118	−1.3	12.34
**N-EtFOSA**	525.97750	525.97668	−1.6	12.66
**FOSAA**	555.95168	555.95148	−0.4	9.47
**N-MePFOSAA**	569.96733	569.96744	−1.0	9.79
**N-EtPFOSAA**	583.98298	583.98285	−0.2	10.06
**N-MeFOSE**	616.00920	616.00842	−1.3	12.32
**N-EtFOSE**	630.02485	630.02405	−1.3	12.63
**6:2 FTCA**	376.98527	376.98499	−0.8	7.51
**8:2 FTCA**	476.97888	476.97894	0.1	8.97
**10:2 FTCA**	576.97249	576.97266	0.3	10.12
**PFHxPA**	398.94612	398.94586	−0.6	5.33
**PFOPA**	498.93973	498.93964	−0.2	7.1
**PFDPA**	598.93335	598.93359	0.4	8.86
**Cl-PFHxPA**	414.91657	414.91641	−0.4	5.58
**Cl-PFOPA**	514.91018	514.91028	0.2	7.38
**PF4OPeA**	228.97411	228.97412	0.05	5.51
**PF5OHxA**	278.97092	278.97092	−0.01	6.23
**NFDHA**	294.96584	294.96588	0.1	6.68
**HFPO–DA**	328.96773	328.96793	0.6	7.08
**HFPO–TA**	494.95306	494.95267	−0.8	9.22
**PFEESA**	314.93791	314.93768	−0.7	6.9
**4:2FTS**	326.97429	326.97421	−0.2	6.57
**6:2FTS**	426.96790	426.96765	−0.6	8.1
**8:2FTS**	526.96152	526.96143	−0.2	9.39
**10:2FTS**	626.95513	626.95465	−0.8	10.39
**8:2diPAP**	988.96228	988.96161	−0.7	11.96
**NaDONA**	376.96887	376.96854	−0.9	7.74
**6:2 Cl-PFESA**	530.89558	530.89520	−0.7	9.64
**8:2 Cl-PFESA**	630.88919	630.88837	−1.3	10.52
**^13^C^4^ PFBA**	216.99262	216.99251	−0.5	5.17
**^13^C^5^ PFPeA**	267.99278	267.99271	−0.3	6
**^13^C^5^ PFHxA**	317.98959	317.98935	−0.8	6.79
**^13^C^4^ PFHpA**	366.98304	366.98248	−1.5	7.57
**^13^C^8^ PFOA**	420.99326	420.99268	−1.4	8.3
**^13^C^9^ PFNA**	471.99343	471.99301	−0.9	8.94
**^13^C^6^ PFDA**	518.98017	518.97974	−0.8	9.52
**^13^C^7^ PFUnDA**	569.98033	569.97998	−0.6	10.03
**^13^C^2^ PFDoA**	614.96036	614.95953	−1.3	10.47
**^13^C^2^ PFTeDA**	714.95397	714.95337	−0.8	11.19
**^13^C^3^ PFBuS**	301.95306	301.95255	−1.7	6.55
**^13^C^8^ PFOS**	506.95706	506.95630	−1.5	9.28
**^13^C^3^ PFHxS**	401.94667	404.94595	−1.8	8.05
**^13^C^8^ FOSA**	505.97304	505.97211	−1.8	11.32
**d3-N-MePFOSAA**	572.98616	572.98578	−0.7	9.79
**d5-N-EtPFOSAA**	589.01436	589.01416	−0.3	10.05
**^13^C^2^ 4:2 FTS**	328.98100	328.98087	−0.4	6.57
**^13^C^2^ 6:2 FTS**	428.97461	428.97543	1.9	8.11
**^13^C^2^ 8:2 FTS**	528.96823	528.96875	1.0	9.39
**^13^C^4^ 8:2 diPAP**	992.97570	992.97510	−0.6	11.96

**Table 2:** The method linear range, instrumental limit of quantification (LOQ), method limit of detection/quantification (MLOD/MLOQ) of 47 target PFASs.

Compound	Linear Range (μg/L)	Correlation Coefficient (R^2^)	LOQ (μg/L)	MLOD (μg/L)	MLOQ (μg/L)
**PFCAs**					
**PFBA**	0.05–25	0.9988	0.05	0.03	0.12
**PFPeA**	0.05–25	0.9986	0.05	0.05	0.2
**PFHxA**	0.01–25	0.9963	0.01	0.02	0.08
**PFHpA**	0.005–25	0.9990	0.005	0.005	0.02
**PFOA**	0.005–25	0.9989	0.005	0.02	0.08
**PFNA**	0.01–25	0.9981	0.01	0.004	0.016
**PFDA**	0.01–25	0.9990	0.01	0.01	0.04
**PFUnDA**	0.05–25	0.9991	0.05	0.005	0.02
**PFDoA**	0.01–25	0.9974	0.01	0.004	0.016
**PFTeDA**	0.05–25	0.9989	0.05	0.02	0.08
**PFSAs**					
**PFBuS**	0.001–25	0.9991	0.001	0.002	0.008
**PFPeS**	0.001–25	0.9987	0.001	0.002	0.008
**PFHxS**	0.001–25	0.9986	0.001	0.002	0.008
**PFOS**	0.001–25	0.9993	0.001	0.003	0.012
**PFNS**	0.001–25	0.9994	0.001	0.002	0.008
**PFDS**	0.001–25	0.9988	0.001	0.002	0.008
**PFDoS**	0.005–25	0.9991	0.005	0.003	0.012
**FOSAs**					
**FOSA**	0.001–25	0.9992	0.001	0.002	0.008
**N–MeFOSA**	0.001–25	0.9985	0.001	0.002	0.008
**N–EtFOSA**	0.001–25	0.9992	0.001	0.008	0.032
**FOSAAs**					
**FOSAA**	0.005–25	0.9928	0.005	0.003	0.012
**N–MePFOSAA**	0.01–25	0.9977	0.01	0.006	0.024
**N–EtPFOSAA**	0.01–25	0.9989	0.01	0.005	0.02
**FOSEs**					
**N–MeFOSE**	0.005–25	0.9976	0.005	0.003	0.012
**N–EtFOSE**	0.005–25	0.9990	0.005	0.003	0.012
**n:2 FTCAs**					
**6:2 FTCA**	0.25–25	0.9980	0.25	0.3	1.2
**8:2 FTCA**	0.1–25	0.9987	0.1	0.05	0.2
**10:2 FTCA**	0.05–25	0.9989	0.05	0.03	0.12
**PFPAs**					
**PFHxPA**	0.05–25	0.9975	0.05	0.03	0.12
**PFOPA**	0.1–25	0.9967	0.1	0.008	0.032
**PFDPA**	0.25–25	0.9938	0.25	0.06	0.24
**Cl–PFHxPA**	0.1–25	0.9976	0.1	0.05	0.2
**Cl–PFOPA**	0.1–25	0.9956	0.1	0.03	0.12
**PFECAs**					
**PF4OPeA**	0.01–25	0.9989	0.01	0.02	0.08
**PF5OHxA**	0.005–25	0.9985	0.005	0.005	0.02
**NFDHA**	0.05–25	0.9983	0.05	0.03	0.12
**HFPO–DA**	0.25–25	0.9911	0.25	0.1	0.4
**HFPO–TA**	0.5–25	0.9915	0.5	0.2	0.8
**PFESAs**					
**PFEESA**	0.005–25	0.9991	0.005	0.005	0.02
**n:2 FTSs**					
**4:2FTS**	0.01–25	0.9995	0.01	0.006	0.024
**6:2FTS**	0.05–25	0.9969	0.05	0.03	0.12
**8:2FTS**	0.01–25	0.9944	0.01	0.004	0.016
**10:2FTS**	0.05–25	0.9948	0.05	0.02	0.08
**diPAPs**					
**8:2diPAP**	0.05–25	0.9991	0.05	0.03	0.12
**Other PFAS**					
**NaDONA**	0.005–25	0.9982	0.005	0.004	0.016
**6:2 Cl–PFESA**	0.005–25	0.9994	0.005	0.005	0.02
**8:2 Cl–PFESA**	0.005–25	0.9980	0.005	0.002	0.008

**Table 3:** Accuracy and precision for 47 target PFASs in surface water (concentration: μg/L).

Compound	Accuracy (%)				Intra-Day Precision (%)				Inter-Day Precision (%)			
	0.05	1	5	20	0.05	1	5	20	0.05	1	5	20
**PFBA**		108	99.2	99.7		3.7	2.8	1.6		3.7	1.7	1.3
**PFHxPA**		61.8	88.3	85.5		2.2	4	0.88		4.9	5.7	4.9
**PF4OPeA**	123		112	105	4.7		2.5	0.63	9.4		8.4	6.4
**Cl-PFHxPA**		63.9	83.3	81.4		1.7	4.2	3.9		6.8	7.1	6.4
**PFPeA**		111	97.6	100		3.5	1.5	0.56		2.9	2.3	0.85
**PF5OHxA**	115		103	101	5		3.5	0.2	3.6		5.2	2.1
**PFBuS**	134		98	99.5	1.5		2.7	0.49	1.3		2.3	0.49
**4:2FTS**	94		97.7	96.1	2.1		3.2	0.51	5.4		2.1	1.4
**NFDHA**		116	106	104		1.9	3.3	2.2		7.3	5.7	5.7
**PFHxA**	151		80.6	84.2	2		2.7	0.21	3.4		1.8	1
**PFEESA**	117		107	100	2.6		0.65	0.25	6.1		5.2	2.5
**HFPO-DA**		95.5	104	105		3.2	2.8	1.8		9.4	8.2	5.8
**PFOPA**		58.6	80.9	79.3		1.5	2.5	2.4		6.9	9.4	8.1
**PFPeS**	113		105	99.5	2.7		0.7	1.3	5.3		4.9	1.7
**Cl-PFOPA**		57.7	81.8	81.6		1	4.5	2.5		9.6	8.9	8.5
**6:2 FTCA**		133	115	113		9.5	5.7	0.33		11	6.5	6.2
**PFHpA**	109		97.7	100	4.6		2	1.2	3.7		2.2	1.3
**NaDONA**	114		113	110	6.1		3.5	1.7	3.6		4	2.6
**PFHxS**	104		97.2	100	1.9		2.6	0.16	1.6		2	0.62
**6:2FTS**		94	84.6	100		2.7	3.1	3.3		2	3	4.3
**PFOA**		103	98.2	98.9		0.1	3.6	0.85		1.1	2.6	0.93
**PFNA**	105		98.6	101	1.1		1.4	3.9	1.7		2.2	2.7
**PFDPA**		75.3	103	108		3.3	3.3	5.2		11	13	11
**8:2 FTCA**		103	95.8	98.1		0.62	2.4	1.2		1.8	3.9	3.7
**HFPO-TA**		71.5	82.8	92		2.7	4.8	3.3		8.4	5.7	5.9
**PFOS**	108		97.8	99.4	1.9		2.3	0.11	1.6		2.3	0.41
**8:2FTS**	102		95	111	3.4		2	2.4	4.8		1.9	8.2
**FOSAA**	80.7		97.3	96.4	1.4		1.8	2.3	5.6		2.6	1.8
**PFDA**	124		97.4	101	2.8		2.4	0.83	3.3		1.9	0.85
**6:2 Cl-PFESA**	114		109	102	3		2.2	0.87	3		3.3	4.4
**N-MePFOSAA**	100		94.8	98.7	2		1.4	0.83	4.9		2.9	0.77
**PFNS**	101		99	100	1.1		3.2	0.67	1.7		2.9	2.3
**PFUnDA**		101	98.4	100		0.75	2.4	1.4		0.83	2.3	0.87
**N-EtPFOSAA**	109		98.6	100	3.8		2.9	0.67	3.4		2.4	1.4
**10:2 FTCA**		114	102	110		2.4	3.6	0.76		5.4	4.5	3.3
**PFDS**	98		95.8	96.8	3.5		1.4	0.79	2.4		3.2	1.9
**10:2FTS**		106	103	123		4.1	4.8	2		4.1	4.2	5.3
**PFDoA**	113		110	98.7	2		2.2	0.6	1.6		1.9	0.47
**8:2 Cl-PFESA**	111		104	104	1		1.9	1.8	2.8		2.7	3.8
**PFDoS**	86		75.5	94.3	6.2		3.1	0.78	5		4.3	2.2
**PFTeDA**		112	106	98.6		1.3	3.5	0.17		1.2	2.5	0.59
**FOSA**	102		98	100	2		2.3	1.4	1.4		2.2	0.74
**8:2diPAP**		89.5	94.6	104	4.1		17	3.5		4	19	3.1
**N-MeFOSE**	100		86.1	96	3.5		2.5	1.4	2.2		3	1.6
**N-MeFOSA**	98		94.4	95.5	3.5		2.6	2.7	3		2.2	2.2
**N-EtFOSE**	90.7		82.4	95	4.6		3.1	2	3.3		2.8	1.9
**N-EtFOSA**	96.7		91.8	96.5	2.4		2.1	1.9	3		2.2	1.4

## Data Availability

Data will be made available on request.

## Supplementary Materials

The following supporting information can be downloaded at: https://www.mdpi.com/article/10.3390/molecules29163881/s1, Figure S1: The responses of target compounds of the 4 columns; Figure S2: The responses of target compounds of the mobile phase gradients; Figure S3: Evaluation of solvent matrix effects; Table S1: List of the 47 target PFASs investigated in this study and their CAS numbers, formulas and corresponding ISs; Table S2: Summary of the specifications and results of the 4 tested columns; Table S3: PFASs concentrations (µg/L) detected in solvent blanks and laboratory procedural blanks; Table S4: Accuracy and precision for 46 target PFASs in ground and waste water (concentration: μg/L); Table S5: PFASs concentrations (μg/L) in ground and waste water samples.
