# Supplementary material for: Rapid Determination of 12 Classes of Per- and Polyfluoroalkyl Substances in Water Samples from Environmental Forensic Cases
Source: Molecules. 2024 Aug 16;29(16):3881. doi: 10.3390/molecules29163881 (PMC11356886; doi:10.3390/molecules29163881)
Supplement: Supplementary file 1 [file molecules-29-03881-s001.zip › molecules-3130883-supplementary.pdf]

Supplementary Information

Rapid determination of 12 classes of per- and polyfluoroalkyl substances in water samples from environmental forensic cases

Bing Li[1, 2], Meihui Wang[1, 2], Kuan Cheng[2], Xueyan Guo[2], Ruyin Dong[2], Keming Yun[1*], Dong Ma[2*]

1 Shanxi Key Laboratory of Forensic Medicine, Key Laboratory of Forensic Toxicology of Ministry of Public Security, School of Forensic Medicine, Shanxi Medical University, Jinzhong, 030600, Shanxi, China

2 Key Laboratory of Forensic Science, Ministry of Justice, Shanghai Forensic Service Platform, Academy of Forensic Science, Shanghai, 200063, China

*Correspondence: yunkeming5142@163.com

*Correspondence: madong@ssfjd.cn

Chemicals

The list of 47 target per- and polyfluoroalkyl substances (PFASs) and 20 isotopically labeled analogues used as internal standards (ISs) with their detailed information is given in Table S1. The PFASs can be classified into 12 classes, i.e., PFCAs (10), PFSAs (7), FOSAs (3), FOSAAs (3), FOSEs (2), n:2 FTCAs (3), PFPAs (5), PFECAs (5), PFESAs (1), n:2 FTSs (4), di PAPs (1), Other PFASs (3). The mixed stock standard solution (1000 μg/L) and internal standard solution (100 μg/L) were prepared with acetonitrile, working standard solution (200, 10, 1, 0.02 μg/L) were prepared with acetonitrile through serial dilution of the stock standard solution.

Table S1. List of the 47 target PFASs investigated in this study and their CAS numbers, formulas and corresponding ISs.

Compound Name	Acronym	CAS Number	Formula	Internal Standard
**Perfluoroalkyl carboxylic acids (PFCAs)**				
Perfluorobutanoic acid	PFBA	375-22-4	$C_4HF_7O_2$	$^{13}C_4$ PFBA
Perfluoropentanoic acid	PFPeA	2706-90-3	$C_5HF_9O_2$	$^{13}C_5$ PFPeA
Perfluorohexanoic acid	PFHxA	307-24-4	$C_6HF_{11}O_2$	$^{13}C_5$ PFHxA
Perfluoroheptanoic acid	PFHpA	375-85-9	$C_7HF_{13}O_2$	$^{13}C_4$ PFHpA
Perfluorooctanoic acid	PFOA	335-67-1	$C_8HF_{15}O_2$	$^{13}C_8$ PFOA
Perfluorononanoic acid	PFNA	375-95-1	$C_9HF_{17}O_2$	$^{13}C_9$ PFNA
Perfluorodecanoic acid	PFDA	335-76-2	$C_{10}HF_{19}O_2$	$^{13}C_6$ PFDA
Perfluoroundecanoic acid	PFUnDA	2058-94-8	$C_{11}HF_{21}O_2$	$^{13}C_7$ PFUnDA
Perfluorododecanoic acid	PFDoA	307-55-1	$C_{12}HF_{23}O_2$	$^{13}C_2$ PFDoA
Perfluorotetradecanoic acid	PFTeDA	376-06-7	$C_{14}HF_{27}O_2$	$^{13}C_2$ PFTeDA
**Perfluoroalkane sulfonic acids (PFSAs)**				
Perfluorobutane sulfonic acid	PFBuS	375-73-5	$C_4HF_9SO_3$	$^{13}C_3$ PFBuS
Perfluoropentane sulfonic acid	PFPeS	630402-22-1	$C_5F_{11}SO_3^-$	$^{13}C_8$ PFOS
Perfluorohexane sulfonic acid	PFHxS	355-46-4	$C_6HF_{13}SO_3$	$^{13}C_3$ PFHxS
Perfluorooctane sulfonic acid	PFOS	1763-23-1	$C_8HF_{17}SO_3$	$^{13}C_8$ PFOS
Perfluorononane sulfonic acid	PFNS	98789-57-2	$C_9F_{19}SO_3^-$	$^{13}C_8$ PFOS

Table S1. Continued.

Compound Name	Acronym	CAS Number	Formula	Internal Standard
Perfluorodecane sulfonic acid	PFDS	335-77-3	$C_{10}HF_{21}SO_3$	$^{13}C_8$ PFOS
Perfluorododecane sulfonic acid	PFDoS	1260224-54-1	$C_{12}F_{25}SO_3^-$	$^{13}C_8$ PFOS
**Perfluorooctane sulfonamides (FOSAs)**				
Perfluoro-1-octanesulfonamide	FOSA	754-91-6	$C_8H_2F_{17}SNO_2$	$^{13}C_8$ FOSA
N-methylperfluoro-1-octanesulfonamide	N-MeFOSA	31506-32-8	$C_9H_4F_{17}SNO_2$	$^{13}C_8$ FOSA
N-ethylperfluoro-1-octanesulfonamide	N-EtFOSA	4151-50-2	$C_{10}H_6F_{17}SNO_2$	$^{13}C_8$ FOSA
**Perfluorooctane sulfonamido acetic acids (FOSAAs)**				
Perfluoro-1-octanesulfonamidoacetic acid	FOSAA	2806-24-8	$C_{10}H_4F_{17}SNO_4$	d3-N-MePFOSAA
N-methyl-perfluorooctane sulfonamido acetic acid	N-MePFOSAA	2355-31-9	$C_{11}H_6F_{17}SNO_4$	d3-N-MePFOSAA
N-ethyl-perfluorooctane sulfonamido acetic acid	N-EtPFOSAA	2991-50-6	$C_{12}H_8F_{17}SNO_4$	d5-N-EtPFOSAA
**Perfluorooctane sulfonamido ethanols (FOSEs)**				
N-(2-hydroxyethyl) -N-methylperfluoro-octanesulfonamide	N-MeFOSE	24448-09-7	$C_{11}H_8F_{17}SNO_3$	$^{13}C_8$ FOSA
N-(2-hydroxyethyl) -N-ethylperfluoro-octanesulfonamide	N-EtFOSE	1691-99-2	$C_{12}H_{10}F_{17}SNO_3$	$^{13}C_8$ FOSA
**n:2 Fluorinated telomer carboxylic acids (n:2 FTCAs)**				
Perfluorohexyl ethanoic acid	6:2 FTCA	53826-12-3	$C_8H_3F_{13}O_2$	$^{13}C_8$ PFOA
Perfluorooctyl ethanoic acid	8:2 FTCA	27854-31-5	$C_{10}H_3F_{17}O_2$	$^{13}C_8$ PFOA
Perfluorodecyl ethanoic acid	10:2 FTCA	53826-13-4	$C_{12}H_3F_{21}O_2$	$^{13}C_8$ PFOA
**Perfluoroalkylphosphonic acids (PFPAs)**				
Perfluorohexylphosphonic acid	PFHxPA	40143-76-8	$C_6H_2F_{13}PO_3$	$^{13}C_5$ PFHxA
Perfluorooctylphosphonic acid	PFOPA	40143-78-0	$C_8H_2F_{17}PO_3$	$^{13}C_5$ PFHxA
Perfluorodecylphosphonic acid	PFDPA	52299-2-6-0	$C_{10}H_2F_{21}PO_3$	$^{13}C_5$ PFHxA
6-Chloroperfluorohexylphosphonic acid	Cl-PFHxPA	NA	$C_6H_2ClF_{12}PO_3$	$^{13}C_5$ PFHxA
8-Chloroperfluorooctylphosphonic acid	Cl-PFOPA	NA	$C_8H_2ClF_{16}PO_3$	$^{13}C_5$ PFHxA
**Perfluoroalkyl ether carboxylic acids (PFECAs)**				

Table S1. Continued.

**Compound Name**	**Acronym**	**CAS Number**	**Formula**	**Internal Standard**
Perfluoro-4-oxapentanoic acid	PF4OPeA	377-73-1	$C_4HF_7O_3$	$^{13}C_8$ PFOA
Perfluoro-5-oxahexanoic acid	PF5OHxA	863090-89-5	$C_5HF_9O_3$	$^{13}C_5$ PFHxA
Perfluoro-3,6-dioxaheptanoic acid	NFDHA	151772-58-6	$C_5HF_9O_4$	$^{13}C_8$ PFOA
Perfluoro-2-methyl-3-oxahexanoic acid	HFPO-DA	13252-13-6	$C_6HF_{11}O_3$	$^{13}C_8$ PFOA
Perfluoro-2,5-dimethyl-3,6-dioxanonanoic acid	HFPO-TA	13252-14-7	$C_9HF_{17}O4$	$^{13}C_8$ PFOA
**Polyfluoroalkyl ether sulfonic acids (PFESAs)**				
Perfluoroethoxyethane sulfonic acid	PFEESA	117205-07-9	$C_4F_9SO_4^-$	$^{13}C_8$ PFOS
**n:2 Fluorotelomer sulfonates (n:2 FTSs)**				
Sodium1H,1H,2H,2H-perfluorohexanesulfonate	4:2FTS	27619-93-8	$C_6H_4F_9SO_3^-$	$^{13}C_2$ 4:2 FTS
Sodium1H,1H,2H,2H-perfluorooctanesulfonate	6:2FTS	27619-94-9	$C_8H_4F_{13}SO_3^-$	$^{13}C_2$ 6:2 FTS
Sodium1H,1H,2H,2H-perfluorodecanesulfonate	8:2FTS	27619-96-1	$C_{10}H_4F_{17}SO_3^-$	$^{13}C_2$ 8:2 FTS
Sodium1H,1H,2H,2H-perfluorododecanesulfonate	10:2FTS	108026-35-3	$C_{12}H_4F_{21}SO_3^-$	$^{13}C_2$ 8:2 FTS
**Polyfluorinated phosphate diesters (diPAPs)**				
Sodium bis(1H,1H,2H,2H-perfluorodecyl)- phosphate	8:2diPAP	NA	$C_{20}H_8F_{34}PO_4^-$	$^{13}C_4$ 8:2 diPAP
**Other PFAS**				
Sodium dodecafluoro-3H-4,8-dioxanonanoate	NaDONA	958445-44-8	$C_7HF_{12}O_4^-$	$^{13}C_8$ PFOA
Potassium 9-chlorohexadecafluoro-3-oxanonane-1-sulfonate	6:2 Cl-PFESA	73606-19-6	$C_8F_{16}ClSO_4^-$	$^{13}C_8$ PFOS
Potassium 11-chloroeicosafluoro-3-oxaundecane-1-sulfonate	8:2 Cl-PFESA	83329-89-9	$C_{10}F_{20}ClSO_4^-$	$^{13}C_8$ PFOS
**Internal standards**				
Perfluoro-n-(^{13}C4)butanoic acid	^{13}C4 PFBA	1017281-29-6	$^{13}C_4HF_7O_2$	
Perfluoro-n-(^{13}C5)pentanoic acid	^{13}C5 PFPeA	2283397-79-3	$^{13}C_5HF_9O_2$	
Perfluoro-n-(1,2,3,4,6-^{13}C5)hexanoic acid	^{13}C5 PFHxA	2328024-54-8	$^{13}C_5CHF_{11}O_2$	
Perfluoro-n-(1,2,3,4-^{13}C4)heptanoic acid	^{13}C4 PFHpA	2328024-55-9	$^{13}C_4C_3HF_{13}O_2$	

Table S1. Continued.

Compound Name	Acronym	CAS Number	Formula	Internal Standard
Perfluoro-n-(^{13}C8)octanoic acid	^{13}C8 PFOA	1350614-84-4	$^{13}C_8HF_{15}O_2$	
Perfluoro-n-(^{13}C9)nonanoic acid	^{13}C9 PFNA	2283397-80-6	$^{13}C_9HF_{17}O_2$	
Perfluoro-n-(1,2,3,4,5,6-^{13}C6)decanoic acid	^{13}C6 PFDA	2328024-56-0	$^{13}C_6C_4HF_{19}O_2$	
Perfluoro-n-(1,2,3,4,5,6,7-^{13}C7)undecanoic acid	^{13}C7 PFUnDA	NA	$^{13}C_7C_4HF_{21}O_2$	
Perfluoro-n-(1,2-^{13}C12)dodecanoic acid	^{13}C2 PFDoA	960315-52-0	$^{13}C_2C_{10}HF_{23}O_2$	
Perfluoro-n-(1,2-^{13}C12)tetradecanoic acid	^{13}C2 PFTeDA	NA	$^{13}C_2C_{12}HF_{27}O_2$	
Sodium perfluoro-1-(2,3,4-^{13}C3)butane sulfonate	^{13}C3 PFBuS	2708218-84-0	$^{13}C_3CHF_9SO_3$	
Sodium perfluoro-1-(^{13}C8)octane sulfonate	^{13}C8 PFOS	2522762-16-7	$^{13}C_8HF_{17}SO_3$	
Sodium perfluoro-1-(1,2,3-^{13}C3)hexane sulfonate	^{13}C3 PFHxS	2708218-86-2	$^{13}C_3C_3HF_{13}SO_3$	
Perfluoro-1-(^{13}C8)octanesulfonamide	^{13}C8 FOSA	NA	$^{13}C_8H_2F_{17}SNO_2$	
N-methyl-d3-perfluorooctane sulfonamido acetic acid	d3-N-MePFOSAA	NA	$C_{11}D_3H_3F_{17}SNO_4$	
N-ethyl-d5-perfluorooctane sulfonamido acetic acid	d5-N-EtPFOSAA	1265205-97-7	$C_{12}D_5H_3F_{17}SNO_4$	
Sodium1H,1H,2H,2H-perfluoro(1,2-^{13}C2)hexanesulfonate	^{13}C2 4:2 FTS	NA	$^{13}C_2C_4H_4F_9SO_3^-$	
Sodium1H,1H,2H,2H-perfluoro(1,2-^{13}C2)octanesulfonate	^{13}C2 6:2 FTS	2708218-89-5	$^{13}C_2C_6H_4F_{13}SO_3^-$	
Sodium1H,1H,2H,2H-perfluoro(1,2-^{13}C2)decanesulfonate	^{13}C2 8:2 FTS	NA	$^{13}C_2C_8H_4F_{17}SO_3^-$	
Sodium bis(1H,1H,2H,2H-(1,2-^{13}C2)perfluorodecyl)-phosphate	^{13}C4 8:2 diPAP	NA	$^{13}C_4C_{16}H_8F_{34}PO_4^-$	

NA: not available

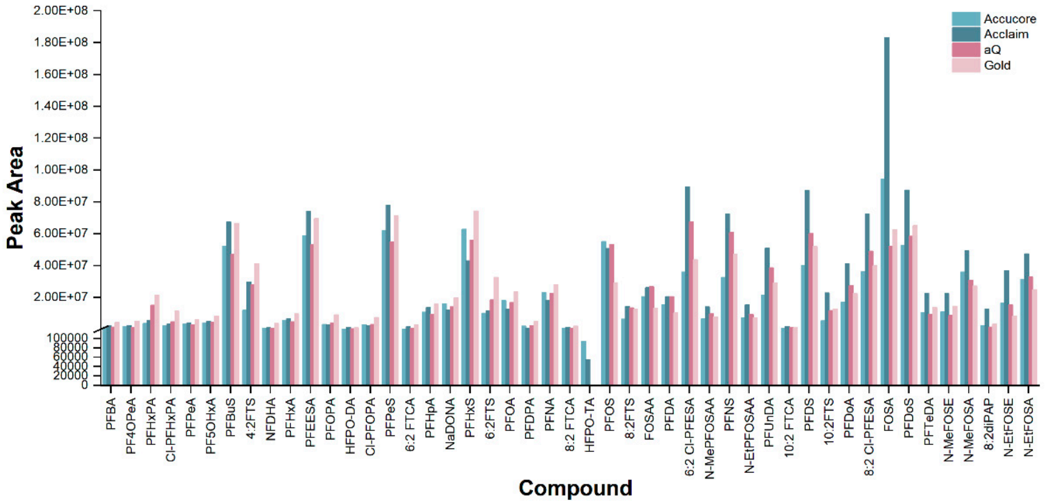

Fig. S1. The responses of target compounds of the 4 columns.

Table S2. Summary of the specifications and results of the 4 tested columns.

Column	Physical dimension (mm)	Particle size (μm)	Pore size (Å)	Surface area (m^2/g)	pH range	Remarks
Accucore	2.1×100	2.6	80	130	1-11	Better separated; not perfect
Acclaim	2.1×150	2.2	120	300	2.5-7.5	Much better separated; selected for this study
aQ	2.1×100	1.7	100	320	2-8	Asymmetric peak shape
Gold	2.1×100	1.9	175	220	2-8	Poor separation

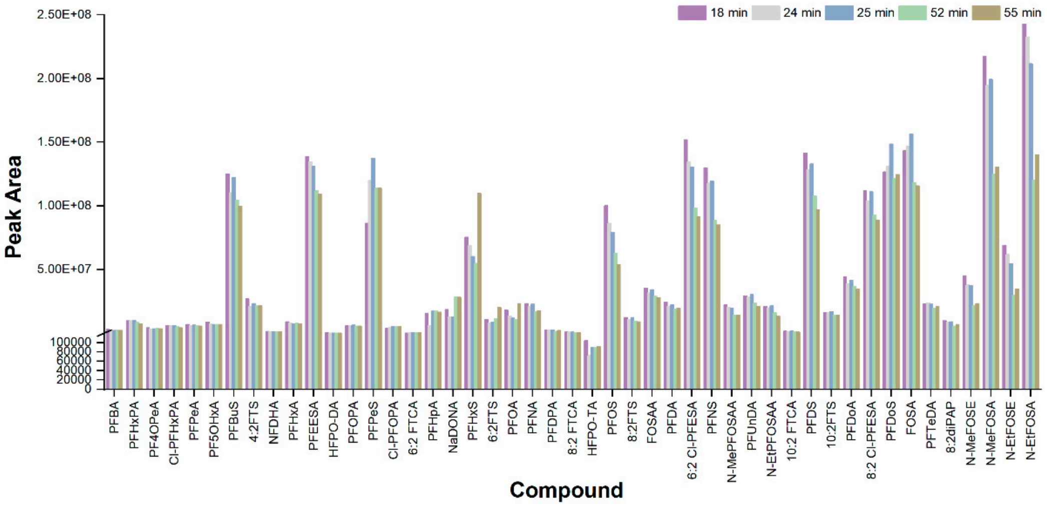

Fig. S2. The responses of target compounds of the mobile phase gradients.

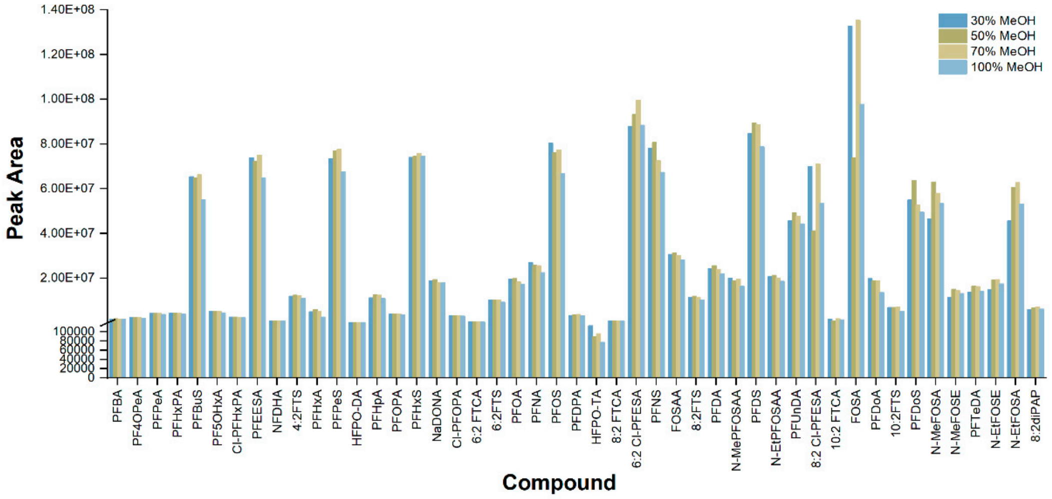

Fig. S3. Evaluation of solvent matrix effects.

Table S3. PFASs concentrations (μg/L) detected in solvent blanks and laboratory procedural blanks.

Compound	Solvent blanks			Laboratory procedure blanks		
	blank-1	blank-2	blank-3	blank-1	blank-2	blank-3
PFBA	<1/3 LOQ	<1/3 LOQ	<1/3 LOQ	<1/3 LOQ	<1/3 LOQ	<1/3 LOQ
PFPeA	<1/3 LOQ	<1/3 LOQ	<1/3 LOQ	<1/3 LOQ	<1/3 LOQ	<1/3 LOQ
PFHxA	<1/3 LOQ	<1/3 LOQ	<1/3 LOQ	<1/3 LOQ	<1/3 LOQ	<1/3 LOQ
PFHpA	ND	ND	ND	ND	ND	ND
PFOA	<1/3 LOQ	<1/3 LOQ	<1/3 LOQ	<1/3 LOQ	<1/3 LOQ	<1/3 LOQ
PFNA	ND	ND	ND	ND	ND	ND
PFDA	<1/3 LOQ	<1/3 LOQ	<1/3 LOQ	<1/3 LOQ	<1/3 LOQ	<1/3 LOQ
PFUnDA	ND	ND	ND	ND	ND	ND
PFDoA	ND	ND	ND	ND	ND	ND
PFTeDA	ND	ND	ND	ND	ND	ND
PFBuS	<1/3 LOQ	<1/3 LOQ	<1/3 LOQ	<1/3 LOQ	<1/3 LOQ	<1/3 LOQ
PFPeS	ND	ND	ND	ND	ND	ND
PFHxS	ND	ND	ND	ND	ND	ND
PFOS	ND	ND	ND	<1/3 LOQ	ND	ND
PFNS	ND	ND	ND	ND	ND	ND
PFDS	ND	ND	ND	ND	ND	ND
PFDoS	ND	ND	ND	ND	ND	ND
FOSA	ND	ND	ND	ND	ND	ND
N-MeFOSA	ND	ND	ND	ND	ND	ND
N-EtFOSA	ND	ND	ND	ND	ND	ND
FOSAA	ND	ND	ND	ND	ND	ND
N-MePFOSAA	ND	ND	ND	ND	ND	ND

Table S3. Continued.

Compound	Solvent blanks			Laboratory procedure blanks		
	blank-1	blank-2	blank-3	blank-1	blank-2	blank-3
N-EtPFOSAA	ND	ND	ND	ND	ND	ND
N-MeFOSE	ND	ND	ND	ND	ND	ND
N-EtFOSE	ND	ND	ND	ND	ND	ND
6:2 FTCA	ND	ND	ND	ND	ND	ND
8:2 FTCA	ND	ND	ND	ND	ND	ND
10:2 FTCA	ND	ND	ND	ND	ND	ND
PFHxPA	ND	ND	ND	ND	ND	ND
PFOPA	ND	ND	ND	ND	ND	ND
PFDPA	ND	ND	ND	ND	ND	ND
Cl-PFHxPA	ND	ND	ND	ND	ND	ND
Cl-PFOPA	ND	ND	ND	ND	ND	ND
PF4OPeA	ND	ND	ND	ND	ND	ND
PF5OHxA	ND	ND	ND	ND	ND	ND
NFDHA	ND	ND	ND	ND	ND	ND
HFPO-DA	ND	ND	ND	ND	ND	ND
HFPO-TA	ND	ND	ND	ND	ND	ND
PFEESA	ND	ND	ND	ND	ND	ND
4:2FTS	ND	ND	ND	ND	ND	ND
6:2FTS	<1/3 LOQ	<1/3 LOQ	<1/3 LOQ	<1/3 LOQ	<1/3 LOQ	<1/3 LOQ
8:2FTS	ND	ND	ND	ND	ND	ND
10:2FTS	ND	ND	ND	ND	ND	ND
8:2diPAP	ND	ND	ND	ND	ND	ND
NaDONA	ND	ND	ND	ND	ND	ND

Table S3. Continued.

Compound	Solvent blanks			Laboratory procedure blanks		
	**blank-1**	**blank-2**	**blank-3**	**blank-1**	**blank-2**	**blank-3**
6:2 Cl-PFESA	ND	ND	ND	ND	ND	ND
8:2 Cl-PFESA	ND	ND	ND	ND	ND	ND

ND: not detected

Table S4. Accuracy and precision for 46 target PFASs in ground and waste water (concentration: μg/L).

Compound	Matrix	Accuracy (%)				Intra-day precision (%)				Inter-day precision (%)			
		0.05	1	5	20	0.05	1	5	20	0.05	1	5	20
PFBA	ground water		113	100	103		2.2	1.5	1.2		2.3	1.3	1.1
	waste water		105	98.3	105		2.5	1.1	6.6		2.8	1.3	6.3
PFHxPA	ground water		52.2	73.7	72.5		2.2	1.5	2.9		4.4	7.4	7.6
	waste water		60.1	88.3	92.3		1	4.3	0.97		4.9	4.4	7.9
PF4OPeA	ground water	125		112	100	5.2		1.6	5.5	6.5		7.2	6.8
	waste water	124		117	108	1.6		7.1	16	11		5.9	11
Cl-PFHxPA	ground water		56.7	76.4	72.8		10	9.3	3.2		9.1	12	9.5
	waste water		64	82.9	82.6		4.6	8.2	3.7		6.6	7.9	10
PFPeA	ground water		116	98	101		3.2	1.2	1.5		3.6	1.2	1.4
	waste water		109	96.9	104		2	1.5	7		1.4	0.9	6.5
PF5OHxA	ground water	119		102	101	8.3		1.6	3.1	6.4		1.5	2.5
	waste water	123		103	106	4.1		2.1	6.6	6.3		2.3	5.5
PFBuS	ground water	149		98	102	4.3		0.36	0.44	2.7		0.64	0.33
	waste water	131		97.6	105	0.88		0.54	5.8	1.8		0.5	5.5
4:2FTS	ground water	101		94.6	98.6	3		0.99	1.2	3.9		1.7	1.3
	waste water	117		97.2	103	4.3		2.4	5.2	6.4		1.6	5.1
NFDHA	ground water		118	104	99.4		2.6	3.3	4.5		5.8	6.4	6.3
	waste water		112	107	107		8.8	6.8	15		6.9	5.3	9.6
PFHxA	ground water	165		82.3	87.7	3.7		3.4	0.98	4.3		2.6	1.3
	waste water	119		49	52.2	2.6		1.7	5.7	3.8		2	5.4
PFEESA	ground water	122		106	102	3.3		3.6	2.4	6.2		7.9	4.8
	waste water	113		104	106	1		11	13	10		6.5	8.4

Table S4. Continued.

Compound	Matrix	Accuracy (%)				Intra-day precision (%)				Inter-day precision (%)			
		**0.05**	**1**	**5**	**20**	**0.05**	**1**	**5**	**20**	**0.05**	**1**	**5**	**20**
HFPO-DA	ground water		100	105	103		0.25	3.7	4.1		9.6	9.8	6
	waste water		92.6	108	109		16	10	18		12	7.5	11
PFOPA	ground water		56.4	76.4	80.8		1.8	1.7	5.3		9.9	13	13
	waste water		61	81	88.2		4.4	8.2	3.2		7.7	8.5	11
PFPeS	ground water	122		105	100	4.3		3.9	1.7	6		6.1	3.5
	waste water	115		104	106	2		8.1	10	7.3		4.9	6.6
Cl-PFOPA	ground water		57.1	77.2	82		2.1	1.7	5.4		9.3	14	13
	waste water		64.4	84.5	92.9		6.3	14	2.8		8.4	12	12
6:2 FTCA	ground water		133	109	108		2.5	4	1.6		6.6	2.8	2.2
	waste water		121	102	106		12	6.2	12		7.6	6	10
PFHpA	ground water	115		96.6	102	2		0.024	1.1	2.7		0.83	0.79
	waste water	111		97.1	105	1		0.95	5.8	2.5		0.96	5.2
NaDONA	ground water	129		113	109	4.5		1.7	2.1	5.1		3.4	2.8
	waste water	115		103	112	2.6		1.9	11	3.8		2.7	7.8
PFHxS	ground water	111		96.7	102	4.5		0.7	0.68	3.3		0.53	0.48
	waste water	123		96.7	106	4.1		0.71	6.1	5.8		0.41	5.6
6:2FTS	ground water		122	91.8	105		20	2.6	3.5		18	2.6	2.4
	waste water		138	95.9	107		3.8	3.1	4.1		3.9	3.2	5.3
PFOA	ground water		110	98.7	101		0.8	0.62	0.61		0.6	0.69	0.43
	waste water		101	96.7	104		1.4	0.17	6.7		0.9	0.6	5.9
PFNA	ground water	115		100	104	2.6		1.4	2.8	1.9		1.1	1.7
	waste water	115		101	112	2		2.7	6.3	2.9		3	5.6
PFDPA	ground water		74.8	105	116		6.8	2.4	8.5		12	17	16

Table S4. Continued.

Compound	Matrix	Accuracy (%)				Intra-day precision (%)				Inter-day precision (%)			
		0.05	1	5	20	0.05	1	5	20	0.05	1	5	20
	waste water		77.4	105	119		5.8	5	3		11	11	14
8:2 FTCA	ground water		107	98.5	101		1.9	3.3	0.33		3.5	2.8	1.6
	waste water		104	88.4	95.2		2.9	1.1	7		2.9	2.9	6.2
HFPO-TA	ground water		75.5	89.9	102		13	2.7	0.65		9.7	5.1	2.8
	waste water		80.8	92.7	103		8.1	2.3	9		7	5.3	9.5
PFOS	ground water	113		97.6	102	2.7		0.63	0.37	3.3		0.63	0.71
	waste water	115		97.8	105	2.7		0.44	6.1	2.2		0.35	5.5
8:2FTS	ground water	109		94.8	102	6.5		1.6	8.5	5		1.2	7.7
	waste water	121		91.7	107	6.2		0.5	1.4	6.2		2	9.4
FOSAA	ground water	91.3		87.2	100	3.3		1.4	1.2	4.8		3.3	3.7
	waste water	104		98.3	115	5.1		2.9	5.2	4.5		3.6	5.9
PFDA	ground water	128		97	102	1.6		1.4	1.3	2.5		0.9	0.92
	waste water	131		97	103	4.7		0.85	6.6	3.3		0.71	5.5
6:2 Cl-PFESA	ground water	121		111	115	3.4		3.2	3.6	3.6		2.7	3.6
	waste water	124		106	116	3.2		1.6	6.2	3.1		2.3	5.8
N-MePFOSAA	ground water	113		94.8	103	2.7		0.44	0.43	2.6		0.61	0.72
	waste water	118		94.3	106	4.5		1.2	5.1	4.3		0.95	5.5
PFNS	ground water	110		99.1	104	4.8		1.7	3.3	4.5		1.8	2.6
	waste water	115		98.4	105	3.6		3.7	6.8	3.2		2.7	5.7
PFUnDA	ground water		101	97.8	102		0.29	1.7	0.61		0.61	1.1	0.74
	waste water		100	98.3	106		1.3	0.61	5.6		1.1	0.67	5.3
N-EtPFOSAA	ground water	113		98	101	4.4		0.73	1.5	3.9		0.97	1.3
	waste water	117		99.4	105	4.3		1.1	5.7	4.3		0.81	5.1

Table S4. Continued.

Compound	Matrix	Accuracy (%)				Intra-day precision (%)				Inter-day precision (%)			
		0.05	1	5	20	0.05	1	5	20	0.05	1	5	20
10:2 FTCA	ground water		120	106	115		6.1	4.8	5.4		4.9	5.3	5.4
	waste water		94.2	45.7	86.5		6	9.3	7.5		5.1	9.1	6.1
PFDS	ground water	105		94	104	4		2.2	2.3	3.5		2.8	2.2
	waste water	107		91.4	104	3.9		3.2	5.5	3.5		2.5	5.4
10:2FTS	ground water		114	99.5	113		9.1	3	9.1		5.6	2.9	8
	waste water		111	102	107		2.2	7.2	1.5		2.8	6.7	7.6
PFDoA	ground water	119		109	100	3.5		0.11	1	4.2		1.1	0.6
	waste water	121		108	102	5		1.1	4.9	3.6		0.81	4.6
8:2 Cl-PFESA	ground water	121		102	116	2.5		1.6	2.8	2.2		3.4	2.2
	waste water	120		97.4	115	6		3.4	7.5	3.9		2.5	5.7
PFDoS	ground water	74.7		59.7	96.2	15		6	4	12		5.9	3
	waste water	70		50.4	91.1	18		19	5.8	15		16	4.8
PFTeDA	ground water		111	107	100		0.63	0.95	0.62		0.64	1.1	0.38
	waste water		111	111	103		1	5.2	4.6		1.6	3.8	4.5
FOSA	ground water	109		97.3	102	6.4		0.64	0.87	3.9		0.54	0.53
	waste water	113		97.5	105	1		0.27	6	2		0.35	5.4
N-MeFOSE	ground water	105		88.5	94.8	2.2		2.2	1	3.1		1.9	2
	waste water	116		91.8	101	4.6		1.8	5.3	3.4		1.5	4.6
N-MeFOSA	ground water	103		92.4	98.7	2.2		2.2	1.6	2.7		2.3	2
	waste water	101		86.4	100	2.3		4.4	4.9	2.6		2.5	4.2
N-EtFOSE	ground water	98		84.5	96.1	4.1		1.7	0.87	2.6		2.3	1.5
	waste water	109		89.3	98.5	4.6		5	4.3	3.6		3.1	4

Table S4. Continued.

Compound	Matrix	Accuracy (%)				Intra-day precision (%)				Inter-day precision (%)			
		**0.05**	**1**	**5**	**20**	**0.05**	**1**	**5**	**20**	**0.05**	**1**	**5**	**20**
N-EtFOSA	ground water	101		89.9	98.4	4.1		2.8	3.1	2.4		3.3	2.8
	waste water	98		82.9	98.9	2		5.9	4.7	3.4		4.4	4.2

Table S5. PFASs concentrations (μg/L) in ground and waste water samples.

	PFBA	PFPeA	PFHxA	PFHpA	PFOA	PFDA	PF4OPeA	PFBuS	PFPeS	PFHxS	PFOS	PFNS	6:2FTCA	6:2FTS	6:2 Cl-PFESA
E-G-1	ND	0.23	0.12	ND	2.21	ND	ND	0.21	0.048	6.01	16.52	ND	ND	5.67	0.82
E-G-2	ND	0.11	0.12	ND	1.5	ND	ND	0.14	0.031	3.83	12.44	ND	ND	3.36	0.22
E-G-3	ND	ND	ND	ND	0.071	ND	ND	0.009	ND	0.013	0.2	ND	ND	ND	ND
E-G-4	ND	ND	ND	ND	0.27	ND	ND	0.02	ND	0.42	7.23	ND	ND	0.38	0.79
L-G-1	ND	ND	ND	ND	0.12	ND	ND	0.023	ND	ND	ND	ND	ND	ND	ND
L-G-2	ND	ND	ND	ND	0.071	ND	ND	0.011	ND	ND	ND	ND	ND	ND	ND
L-G-3	ND	ND	ND	ND	0.1	ND	ND	0.034	ND	ND	0.021	ND	ND	ND	ND
L-G-4	ND	ND	ND	ND	0.088	ND	ND	0.031	ND	ND	0.017	ND	ND	ND	ND
E-W-1	ND	ND	ND	0.043	0.058	ND	ND	0.35	0.92	7.26	15.62	0.014	ND	ND	0.029
E-W-2	2.77	1.57	1.36	0.38	0.056	ND	ND	0.14	0.014	3.15	102.9	ND	ND	ND	0.74
E-W-3	0.82	ND	ND	ND	0.049	ND	ND	0.057	ND	0.023	0.021	ND	ND	ND	ND
E-W-4	ND	ND	ND	ND	ND	ND	ND	0.014	0.063	0.61	80.93	0.014	ND	ND	ND
E-W-5	0.38	ND	0.05	ND	0.65	ND	ND	0.046	0.009	1.03	240.93	0.49	ND	ND	18.87
E-W-6	ND	ND	0.15	0.069	0.17	ND	ND	0.039	0.018	0.54	18.64	ND	ND	136.44	0.17
E-W-7	ND	ND	0.11	0.021	0.11	ND	ND	0.026	ND	0.064	1.79	ND	ND	8.66	ND
L-W-1	ND	ND	ND	ND	0.072	ND	ND	0.021	ND	ND	ND	ND	ND	ND	ND
L-W-2	ND	ND	ND	ND	0.17	0.03	1.66	0.05	ND	ND	ND	ND	ND	ND	ND
L-W-3	ND	ND	ND	ND	0.099	ND	0.22	0.031	ND	ND	ND	ND	2.62	ND	ND
L-W-4	ND	ND	0.038	ND	0.14	ND	ND	0.057	ND	ND	ND	ND	ND	ND	ND
L-W-5	ND	ND	ND	ND	0.091	ND	0.61	0.038	ND	ND	0.013	ND	ND	ND	ND

ND: not detected
